# Effect of crop rotational position and nitrogen supply on root development and yield formation of winter wheat

**DOI:** 10.3389/fpls.2023.1265994

**Published:** 2023-10-23

**Authors:** Jessica Arnhold, Dennis Grunwald, Andrea Braun-Kiewnick, Heinz-Josef Koch

**Affiliations:** ^1^ Department of Agronomy, Institute of Sugar Beet Research, Göttingen, Germany; ^2^ Institute for Epidemiology and Pathogen Diagnostics, Julius Kühn-Institute - Federal Research Centre for Cultivated Plants, Braunschweig, Germany

**Keywords:** root length density, grain yield, nitrogen uptake, biomass, oilseed rape, take-all disease

## Abstract

The lower yield of wheat grown after wheat (second wheat) compared with the first wheat after a break crop is frequently attributed to fungal disease occurrence, but has also been found without visible disease infection; thus, other factors might be responsible for the lower yield of the second wheat. The aims of this study were to analyze the effects of growing wheat as first and second wheat after oilseed rape, as well as monoculture in a long-term field experiment over three years on (i) aboveground biomass formation, root development and nutrient acquisition during the growing season, (ii) take-all occurrence, and (iii) grain yield and yield components. Subsoil root length density of winter wheat was significantly higher after oilseed rape as pre-crop than after wheat. Differences in wheat aboveground biomass occurred at early growth stages and were persistent until harvest. Grain yield loss correlated well with take-all disease severity in a wet year but yield differences among crop rotational positions occurred also in a dry year without visible fungal infection. Thus, an effect of the crop rotational position of wheat beyond take-all disease pressure can be assumed. Overall, wheat root length density might be the key to understand wheat biomass formation and grain yield in different crop rotational positions.

## Introduction

Wheat (*Triticum aestivum* L.) is one of the most important staple food crops worldwide and has been the most cultivated crop in Germany for many years. Here, the crop’s winter type is currently grown on more than 2.8 million ha (Destatis, 2023) and worldwide on more than 220 million ha in 2021 (FAOSTAT, 2023). In Europe, wheat grain yields substantially increased for decades but since the 1990s, yields have stagnated (Brisson et al., 2010).

The most common cause for this stagnation in yield is assumed to be an infection of the wheat plants with the fungus *Gaeumannomyces tritici* (Ggt), the pathogen of the take-all disease, and a subsequent reduction of water and nutrient uptake due to an accelerated root senescence. Infection with Ggt is known to be amplified by repeated cultivation of host plants, and consequently, Weiser et al. (2018) pointed to an increase in wheat cultivation after wheat as, among others, a likely cause for the stagnation in yield. One important positive effect of oilseed rape as pre-crop before wheat is that it is a non-host plant for Ggt and therefore interrupts the pest life cycle as a break crop (Kirkegaard et al., 2008). Cunfer et al. (2006) observed a large effect of oilseed rape in suppressing take-all disease severity in a study with wheat in 12 double-cropping sequences: the yield of wheat grown after oilseed rape was similar to control plots without any take-all occurrence. As another benefit, Sieling et al. (2005) found a reduced susceptibility towards drought stress of wheat grown after oilseed rape compared to wheat grown after wheat.

Another option to cope with yield reductions caused by take-all occurrence might be continuous cultivation of wheat since wheat monoculture can lead to the so-called take-all decline effect, addressing a low, or even undetectable, take-all disease severity after several years of wheat cultivation in a row; nevertheless, grain yield remained lower compared to wheat grown in crop rotation (Cook, 2003). However, even without severe Ggt infection, wheat yield was reduced when grown after wheat, raising the question if other causes contribute to wheat yield decline (Sieling et al., 2007).

Several studies reported beneficial effects of pre-crops on root growth of the following crop, e.g., Monaci et al. (2017) found a higher rootability and root density as well as thinner roots of winter wheat when grown after alfalfa compared to after barley. Furthermore, Perkons et al. (2014) observed a higher root length density especially in the subsoil for winter barley grown after 2 years of chicory compared to after oats or fescue. Similarly, Han et al. (2015) noted a higher root length for spring wheat grown after chicory compared to tall fescue, and Gaiser et al. (2012) found that spring wheat grown after lucerne led to a higher rooting depth and root length density in the subsoil than after chicory and fescue.

In consequence, besides take-all occurrence, such pre-crop specific effects on the root system might play a role in wheat yield, since a less developed root system might limit the water and nutrient uptake of plants (Perkons et al., 2014; Tracy et al., 2020). Perkons et al. (2014) found that crops were able to root deeper and build up a bigger root system after pre-crops with a taproot compared to pre-crops forming fine roots only, due to the presence of wider biopores. Cereals profited from this effect in particular and were able to take up nutrients and water from deeper soil layers at early growth stages (Han et al., 2015). In addition, rootability can be modified by soil structural properties, which may also be affected by other crops grown in the rotation and the rotational position of wheat (Ball et al., 2005). Schönhammer and Fischbeck (1987b) observed in their study in Southern Germany that increasing soil porosity and decreasing penetration resistance after oilseed rape as pre-crop compared to oat or wheat improved root density in subsequent winter wheat and consequently increased yield (Schönhammer and Fischbeck, 1987a). In addition, Sieling et al. (2005) hypothesized that the early root system might be of high importance for later stages of wheat plant growth of wheat grown after wheat.

Thus, the root systems of wheat grown in different crop rotational positions are likely to differ, which can be of high importance for yield formation, independent from the presence or absence of a take-all infection. Commercial farmers in Germany prefer to cultivate winter wheat after oilseed rape to benefit from the yield gain provided by this crop; nevertheless, wheat is frequently grown after wheat due to the generally high profitability of wheat compared to other crops (Kirkegaard et al., 2008; Steinmann and Dobers, 2013; Weiser et al., 2018). Although some knowledge exists, comprehensive studies on wheat above- and belowground biomass development, clearly identifying the cause-and-effect mechanisms in the interplay of root growth and yield formation, after the most common economically important pre-crops wheat and oilseed rape are scarce. More specifically, the response of wheat grown in different crop rotational positions, such as, (i) after oilseed rape, (ii) after wheat grown after oilseed rape break crop (stubble wheat) and (iii) under long-term continuous wheat cultivation (monoculture), has not yet been quantified at the same site to our knowledge.

Thus, in our study we elucidated the effects of first and second wheat after oilseed rape and wheat monoculture in a long-term field experiment on (i) above-ground biomass formation, root development and nutrient acquisition during the growing season, (ii) take-all occurrence, and finally (iii) grain yield and yield components.

## Materials and methods

### Field site and experimental design

The field site is located near Harste (51°36’23.5”N, 9°51’55.8”E, 142 m above sea level) in Central Germany and the soil type is a silty loam Luvisol derived from Loess (IUSS Working Group WRB, 2015). Long-term (1991–2020) mean annual precipitation is 624 mm and mean annual temperature is 9.4°C (DWD, 2022).

The crop rotation trial was established in 2006 (Koch et al., 2018) and includes nine crop rotations, out of which two with winter wheat were included in this study: (1) wheat monoculture (WM) and (2) winter oilseed rape–winter wheat–winter wheat–grain pea–sugar beet–winter wheat. From the latter, the first (W1) and second wheat (W2) after oilseed rape as break crop were considered. Each crop rotation element is cultivated every year and each plot is replicated three times in incomplete blocks. Within plots, mineral N fertilization was varied as a second factor by applying 0 kg N ha^−1^ (N_0_) and approximately 240 kg N ha^−1^ (N_opt_), the latter to provide a total of 265 kg N ha^−1^ including soil mineral N content in spring. For this, the main plot of 227 m² (14 × 16.2 m) was split up into two sub-plots resulting in a split-plot design with the crop rotational position on main level and the N fertilization on sub-plot level.

After winter oilseed rape and winter wheat harvest, reduced soil tillage was performed with a disk harrow to 4 cm soil depth. Before wheat sowing, non-inversion soil tillage was conducted with a cultivator (double-heart coulters; 30 cm distance) to 12 cm soil depth. Winter wheat was sown on 25, 13, and 18 October in 2019, 2020, and 2021, respectively. The cultivar was “Nordkap” (SAATEN-UNION GmbH, Isernhagen, Germany) and sowing density was 375 seeds m^−2^ in 2019 and 400 seeds m^−2^ in 2020 and 2021. Crop management followed the recommendations of the regional extension services, partially adapted according to personal expertise. Weather conditions during the study years were monitored with an on-site weather station that recorded precipitation and temperature hourly.

### Root sampling

Roots of winter wheat were sampled at growth stage BBCH 29 (mid- to end of April) in 2021 and 2022 and at BBCH 69 (mid- to end of June) in 2020–2022. Three soil cores per plot with a diameter of 60 mm were randomly taken using a tractor-mounted hydraulic probe in the wheat rows down to a depth of 120 cm and divided into depths of 0–15, 15–30, 30–60, 60–90, and 90–120 cm. The maximum depth of 120 cm was not reached for each soil core due to technical limitations.

Samples from 0–15 cm were not analyzed at the BBCH 69 sampling date, due to an exceedingly large amount of root and non-root organic material. Roots were washed out of the soil cores with water, manually cleaned from straw and plant residues, and placed on a glass plate. Glass plates were scanned (Epson Perfection V850 Pro, Epson, Suwa, Japan) and analyzed for total root length with the software WinRHIZO 2019 (Regent Instruments, Quebec, Canada). Root length density (RLD) was calculated by referring to the sample volume using the following formula:


$$\text{RLD}\;\left[{\text{cm cm}}^{-3}\right]=\frac{\text{root length [cm]}}{{\text{sample volume [cm}}^{3}]}$$

### Aboveground biomass sampling

Wheat aboveground biomass was sampled at the same dates as the roots, i.e., growth stages BBCH 29 and 69, and in addition at BBCH 59 (end of May) in 2020–2022. Plants were cut off right above the ground with an electric cutter in two areas of 0.5 m² each per plot at all mentioned growth stages. Final harvest took place at growth stage BBCH 93, at which an area of 21 m² was combine-harvested and wheat grain and straw dry matter were determined. For this, a mixed subsample was dried at 105°C for 24 h to determine dry matter content in order to calculate dry matter biomass (dm). Ear density was determined by counting eight plant rows of 1 m length per harvest plot; thousand grain weight was determined by weighing. From these parameters, the number of grains per ear was calculated. In addition, harvest index was calculated by dividing grain yield by total wheat aboveground biomass. Both grain and straw samples were analyzed for total N with the FlashSmart Elemental Analyzer (ThermoFisher Scientific, USA). Whole plant N uptake was calculated by multiplying total N and dry matter biomass. Absolute and relative growth rates between biomass sampling dates were calculated using the following formulas:


$$\text{Absolute growth rate}\;{\text{[kg ha}}^{-1}{\text{d}}^{-1}\text{] =}\frac{{\text{biomass}}_{2}-{\text{biomass}}_{1}}{{\text{t}}_{2}-{\text{ t}}_{1}},$$

$$\text{Relative growth rate }\left[{\text{d}}^{-1}\right]\text{= }\frac{\text{Ln (}\frac{{\text{biomass}}_{2}}{{\text{biomass}}_{1}})}{{\text{t}}_{2}-{\text{t}}_{1}}$$

where t is the timepoint and indices 1 and 2 refer to beginning and end of time periods.

### Take-all disease rating

In 2021 and 2022, the proportion of blackened wheat roots was determined visually after harvest on 100 plants per crop rotational position and N level. Wheat plants with roots were dug up with a spade, and roots were cleaned from soil with water and visually rated at a scale of 0–100% blackened roots in 10%-steps. Afterwards, the take-all-index (TAI) was calculated as:


$$\text{TAI}=\frac{0\text{a}+10\text{b}+30\text{c}+60\text{d}+100\text{e}}{\text{T}},$$

where a, b, c, d, and e are the number of plants rated with 0%, 1%–10%, 11%–30%, 31%–60%, and 61%–100%, respectively, and T is the total number of plants (EPPO, 2008).

To confirm Ggt infection beyond visual assessment, wheat root samples used for disease rating were ground in liquid nitrogen using mortar and pestle. Total DNA was extracted with the CTAB/β-mercaptoethanol method (Allen et al., 2006). Ggt DNA in the root samples was qualitatively detected using a Ggt-specific TaqMan Real-time PCR. The Real-time PCR assays were based on the amplification of a Ggt-specific 106-bp fragment of the translation elongation factor 1-alpha (EF1-alpha) gene with the primers GgtEFF1 and GgtEFR1 as well as the TaqMan MGB probe GgtEFPR1 developed by Keenan et al. (2015) with slight modifications. Briefly, Real-time PCR was performed in reaction volumes of 20 μL containing 1 μL of template DNA, 600 nM of each primer, 300 nM of probe, and 10 μL of BioRAD SsoAdvanced Universal Probes Supermix (2x) (Bio-Rad Laboratories, Inc, USA) for the use on the BIO-RAD CFX Connect™ Real-Time System (Bio-Rad Laboratories GmbH, München, Germany). Cycling conditions were 3 min at 95°C, followed by 40 cycles of 15 s at 94°C, 20 s at 52°C, and 20 s 72°C. All TaqMan Real-time PCR reactions were performed in duplicate.

### Statistical analyses

The statistical data analysis was conducted with R version 4.2.3 (R Foundation for Statistical Computing, Vienna, Austria). Differences between the crop rotational positions regarding RLD, wheat aboveground biomass, N uptake, absolute growth rate, grain yield, ear density, grains ear^−1^, thousand grain weight, and harvest index were analyzed by a linear mixed-model ANOVA with year, crop rotational position, N level, and all two-way interactions, as well as replication and the interaction of replication and year as fixed effects and plot as well as sub-plot as random effects. In a first analysis the three-way interaction was included in the linear model, but the interaction was not significant in any case. Thus, the three-way interaction was excluded from the final linear model. All linear mixed models were calculated with the package “lmerTest”. The residuals of the models were checked for normal distribution graphically and with the Shapiro–Wilk test, and for homoscedasticity with the Levene’s test as well as graphically. The RLD values in 0–15 cm soil depth at BBCH 29 were log-transformed as no homoscedasticity was given. If the factor crop rotational position, the interactions of year and crop rotational position, or crop rotational position and N level were significant (*p*< 0.05), means were compared by a *post-hoc* Tukey test with the package “emmeans”. The relationship between grain yield and TAI was analyzed with a linear regression model by SigmaPlot (version 14.5, Systat Software Inc., USA).

## Results

**Figure 1:**
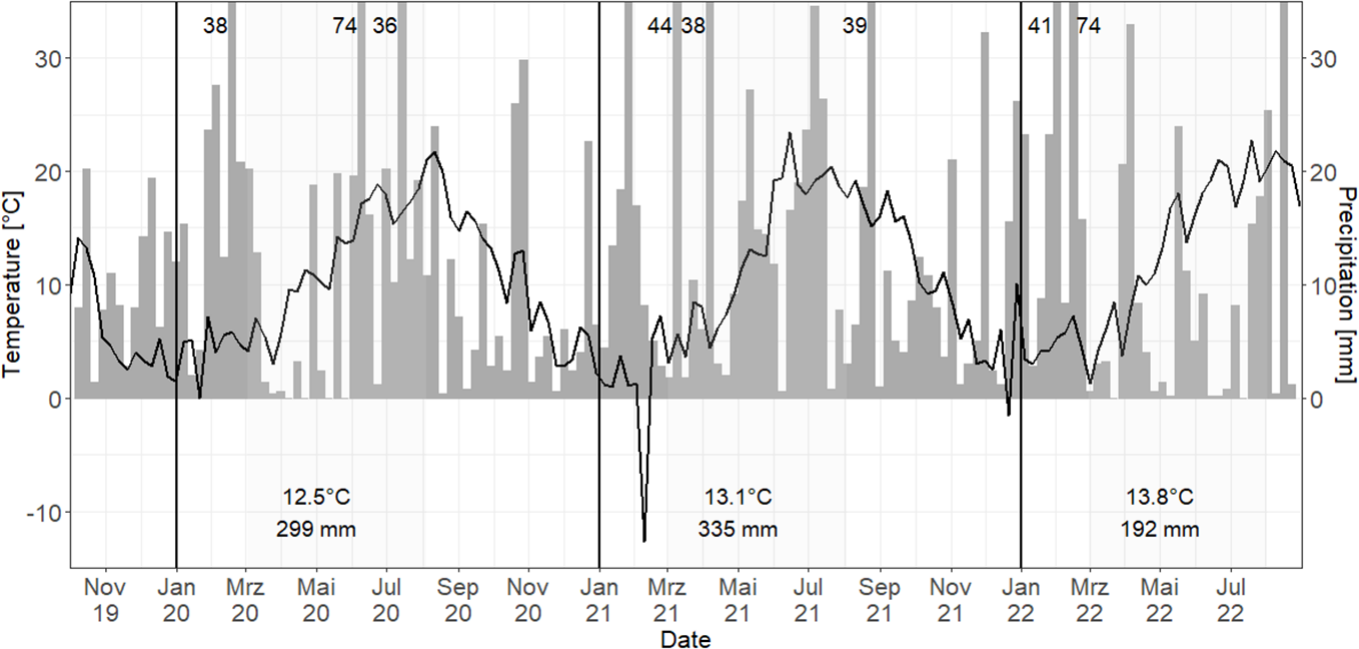
Weekly mean air temperature (°C, black line) and sum of precipitation (mm, gray bars) for the study years 2020–2022 in Harste. Black vertical line = separation of years; gray areas = vegetation periods with mean air temperature and precipitation sum; numbers = precipitation sum > 35 mm.

Among the study years, 2021 had the highest and most evenly distributed precipitation sum over the vegetation period (March–August, **Figure 1**), while in 2020, total annual precipitation was similarly high, but distribution was very uneven with low rainfall in April–June. 2022 had the lowest precipitation and highest weekly mean air temperature.

The factors year and N level had a significant effect on most analyzed parameters, while their interaction was significant for RLD at BBCH 69 in 15–30 cm soil depth, grain yield, and yield components only (**Table 1**). Furthermore, the crop rotational position significantly affected most analyzed parameters, except RLD at BBCH 29 in all soil depths, absolute growth rate in BBCH 59–69, ear density, and harvest index. A significant interaction between study year and crop rotational position was found for RLD at BBCH 69 in 15–30 cm soil depth, aboveground biomass at BBCH 93, wheat grain yield and thousand grain weight. Likewise, a significant interaction occurred between crop rotational position and N level on aboveground biomass and N uptake at BBCH 69 and BBCH 93, absolute growth rate in BBCH 59–69, and wheat grain yield.

**Table 1:** *p*-values for the effects of year (Y), crop rotational position (Crp; W1, W2, WM) and N fertilization (N; N_0_, N_opt_) and their interactions on root length density, wheat aboveground biomass, N uptake, absolute growth rate, wheat grain yield, and wheat yield components; ns, not significant (*p*-value ≥ 0.05).

		Y	Crp	N	Y × Crp	Y × N	Crp × N
**Root length density** **BBCH 29** **[cm cm^−3^]**	0–15 cm	0.0002	ns	ns	ns	ns	ns
	15–30 cm	0.0326	ns	ns	ns	ns	ns
	30–120 cm	ns	ns	ns	ns	ns	ns
**Root length density** **BBCH 69** **[cm cm^−3^]**	15–30 cm	<0.0001	0.0036	0.0007	0.0002	0.0016	ns
	30–120 cm	0.0027	0.0064	ns	ns	ns	ns
**Wheat aboveground biomass** **[Mg dm ha^−1^]**	BBCH 29	<0.0001	0.0234	ns	ns	ns	ns
	BBCH 59	0.0101	0.0061	<0.0001	ns	ns	ns
	BBCH 69	ns	0.0023	<0.0001	ns	ns	0.0088
	BBCH 93	0.0002	<0.0001	<0.0001	0.0123	ns	0.0308
**N uptake** **[kg N ha^−1^]**	BBCH 29	<0.0001	0.0301	0.0001	ns	ns	ns
	BBCH 59	0.0066	0.0484	<0.0001	ns	ns	ns
	BBCH 69	ns	0.0235	<0.0001	ns	ns	0.0213
	BBCH 93	0.0010	0.0015	<0.0001	ns	ns	0.0443
**Absolute growth rate** **[kg dm ha^−1^ d^−1^]**	BBCH 29–59	0.0005	0.0164	<0.0001	ns	ns	ns
	BBCH 59–69	0.0028	ns	0.0010	ns	ns	0.0138
	BBCH 69–93	0.0020	0.0202	<0.0001	ns	ns	ns
**Wheat grain yield** **[Mg dm ha^−1^]**		<0.0001	<0.0001	<0.0001	0.0021	0.0320	0.0242
**Wheat yield components**	Ear density [ears m^−2^]	0.0006	ns	<0.0001	ns	0.0244	ns
	Grains/ear	0.0003	0.0048	<0.0001	ns	0.0067	ns
	Thousand grain weight [g]	<0.0001	0.0021	<0.0001	0.0490	0.0116	ns
	Harvest index	<0.0001	ns	0.0018	ns	0.0143	ns

**Figure 2:**
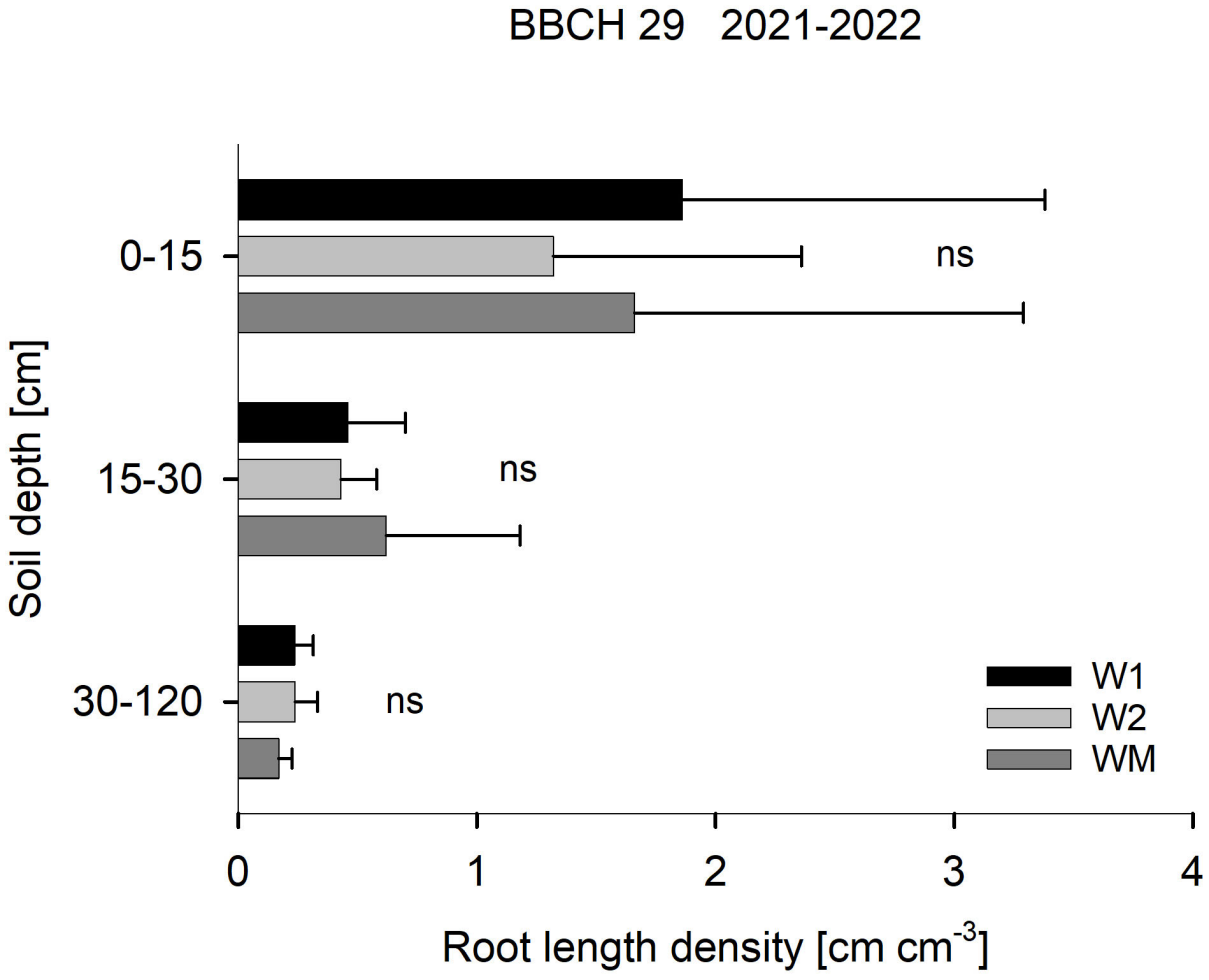
Effect of the crop rotational position of winter wheat (W1 = first wheat after oilseed rape, W2 = second wheat after oilseed rape, WM = wheat monoculture) on root length density in three soil depths at BBCH 29 in Harste, data from 2021 and 2022, *n* = 12. Bars show means with standard deviation, ns, not significant (*p* ≥ 0.05).

**Figure 3:**
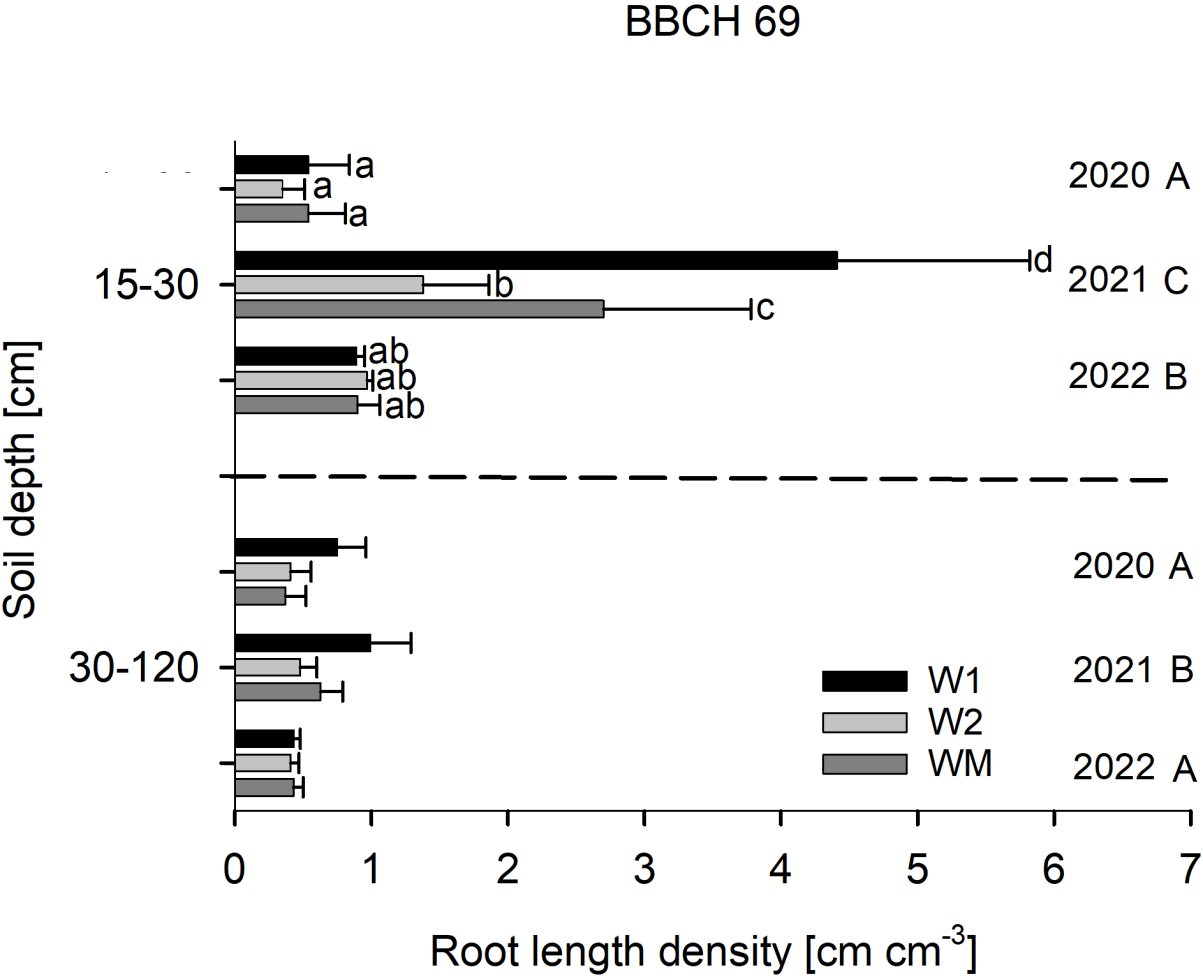
Effect of the crop rotational position of winter wheat (W1 = first wheat after oilseed rape, W2 = second wheat after oilseed rape, WM = wheat monoculture) on root length density (RLD) in two soil depths at BBCH 69 in Harste, n = 6, mean across N fertilization. Bars show means with standard deviation. Lowercase letters show significant differences between means in each depth and capital letters show significant differences between means of each year (p < 0.05). RLD varied significantly with W1 (0.7 ± 0.3 cm cm^−3^) > WM (0.5 ± 0.2 cm cm^−3^) and W2 (0.4 ± 0.1 cm cm^−3^) in 30–120 cm soil depth.

RLD at BBCH 29 was not affected by the crop rotational position (**Figure 2**), RLD in 15–30 cm soil depth at BBCH 69 varied significantly with W1 > WM > W2 in 2021, while no significant differences were found in 2020 and 2022 (**Figure 3**). In 30–120 cm soil depth at BBCH 69, RLD varied significantly with W1 (0.7 ± 0.3 cm cm^−3^) > WM (0.5 ± 0.2 cm cm^−3^) and W2 (0.4 ± 0.1 cm cm^−3^) across all study years (**Figure 3**). For all BBCH stages and soil depths, RLD varied significantly with 2021 > 2022 and, if sampled, 2020 (BBCH 29: data not shown, BBCH 69: **Figure 3**).

At all sampling dates and growth periods, aboveground biomass, N uptake, and absolute growth rate, respectively, were higher for W1 than for W2 and WM, which was significant in most cases (**Table 2**). Across all crop rotational positions, relative growth rate varied between 0.04 ± 0.01 d^−1^ and 0.05 ± 0.01 d^−1^ at BBCH 29–59, between 0.02 ± 0.02 d^−1^ and 0.03 ± 0.01 d^−1^ at BBCH 59–69, and was 0.00 ± 0.00 d^−1^ at BBCH 69–93 (data not shown). For N uptake at BBCH 29 and 93, and absolute growth rate at BBCH 29–59 and 69–93, however, WM was intermediate between W1 and W2, or similarly high as W1. The significant interaction occurring between year and crop rotational position for aboveground biomass at BBCH 93 was caused by a significantly lower value for W2 compared to WM in 2021, but similar biomass in the other study years (**Table 2**). The interaction between crop rotational position and N level for wheat aboveground biomass and N uptake at BBCH 69 and 93 was significant due to similar results for W2 and WM at N_opt_, but lower values at N_0_ (**Table 2**). For the absolute growth rate during BBCH 59–69, significant differences among crop rotational positions occurred at N_opt_ (W1 = W2 > WM), but not at N_0_.

**Table 2:** Effect of the crop rotational position of winter wheat (W1 = first wheat after oilseed rape, W2 = second wheat after oilseed rape, WM = wheat monoculture) and, if significant, the interaction of crop rotational position and N fertilization, as well as crop rotational position and year, on wheat aboveground biomass, N uptake, absolute growth rate, ear density, grains per ear, thousand grain weight, and harvest index in Harste, data from 2020 –2022.

			Crop rotational position		
		Interaction	W1	W2	WM
**Wheat aboveground biomass** **[Mg dm ha^−1^]**	BBCH 29	–––* ^1^	1.0 (0.3) b	10.7 (0.3) a	10.8 (0.3) a
	BBCH 59	–––	7.9 (1.6) b	6.5 (1.7) a	7.1 (1.5) a
	BBCH 69	N_0_ * ^2^	9.4 (0.9) b	7.4 (0.8) a	8.8 (1.4) b
		N_opt_	14.2 (1.5) d	12.1 (0.9) c	11.1 (1.5) c
	BBCH 93	2020* ^3^	13.0 (3.7) cd	11.0 (3.6) ab	11.4 (3.2) ab
		2021	16.6 (5.0) f	10.6 (3.4) a	12.9 (3.4) de
		2022	15.0 (4.3) e	12.0 (4.3) bc	12.9 (3.8) cd
		N_0_	11.0 (1.2) c	7.8 (0.5) a	9.7 (1.9) b
		N_opt_	18.8 (2.2) e	14.6 (1.2) d	15.3 (1.4) d
**N uptake** **[kg N ha^−1^]**	BBCH 29	–––	28 (8) b	22 (9) a	24 (7) ab
	BBCH 59	–––	133 (66) b	116 (62) a	134 (68) b
	BBCH 69	N_0_	88 (12) b	68 (11) a	89 (23) b
		N_opt_	235 (26) d	206 (29) c	199 (19) c
	BBCH 93	N_0_	95 (17) b	74 (13) a	93 (25) b
		N_opt_	249 (24) d	209 (33) c	225 (19) c
**Absolute growth rate** **[kg dm ha^−1^ d^−1^]**	BBCH 29–59	–––	137 (33) b	115 (33) a	126 (31) ab
	BBCH 59–69	N_0_	185 (75) ab	156 (65) a	180 (112) ab
		N_opt_	341 (165) c	273 (79) bc	163 (151) a
	BBCH 69–93	–––	61 (49) b	28 (31) a	48 (45) ab
**Ear density [ears m^−2^]**		–––	460 (94) a	436 (85) a	461 (94) a
**Grains ear^−1^**		–––	35 (5) b	30 (6) a	32 (5) a
**Thousand grain weight [g]**		2020	45 (1) d	43 (2) c	43 (1) c
		2021	40 (2) b	35 (2) a	39 (2) b
		2022	44 (2) cd	43 (2) c	43 (1) c
**Harvest index**		–––	0.48 (0.1) a	0.48 (0.1) a	0.49 (0.0) a

*^1^–––: no interaction, n = 18.

*^2^Interaction crop rotational position and N fertilization, N_0_: without N fertilization, N_opt_: with optimal N fertilization, mean across years, n = 9.

*^3^Interaction crop rotational position and year, mean across N fertilization, n = 6. Mean with standard deviation in brackets. Lowercase letters show significant differences between means within the respective groups (p< 0.05).

**Figure 4:**
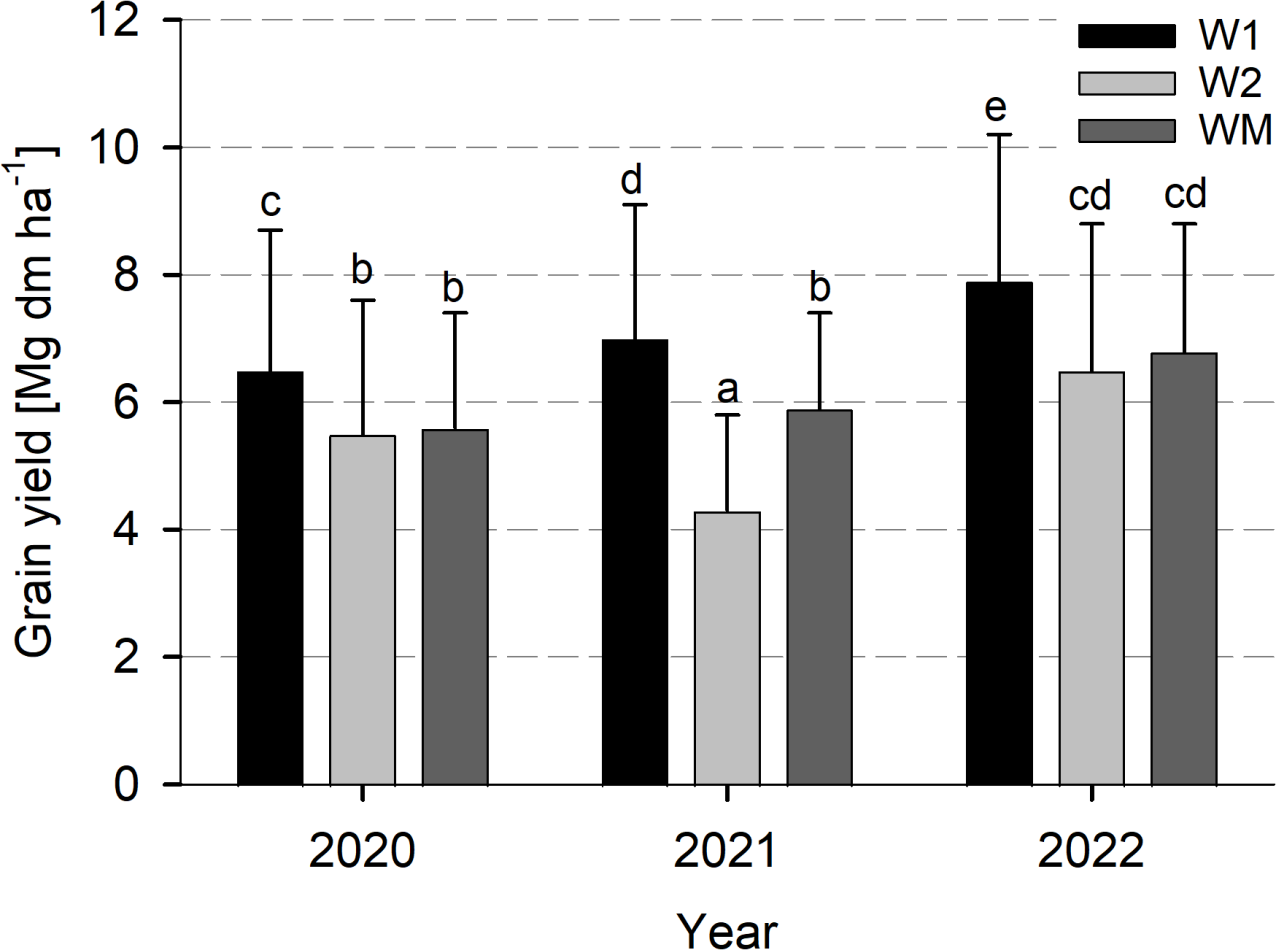
Effect of the crop rotational position of winter wheat (W1 = first wheat after oilseed rape, W2 = second wheat after oilseed rape, WM = wheat monoculture) on wheat grain yield in years 2020, 2021, and 2022 in Harste, *n* = 6, mean across N fertilization. Bars show means with standard deviation. Lowercase letters show significant differences between means (*p*< 0.05).

Wheat grain yield varied significantly with W1 (7.1 ± 2.2 Mg dm ha^−1^) > WM (6.1 ± 1.8 Mg dm ha^−1^) > W2 (5.4 ± 2.1 Mg dm ha^−1^) across all study years (data not shown). For the study years, grain yield varied significantly with 2022 > 2020 and 2021 (data not shown). The significant interaction between year and crop rotational position was caused by a significantly lower value for W2 compared to WM in 2021, but similar grain yield in the other study years (**Figure 4**). The significant interaction between crop rotational position and N level was not caused by contrasting crop rotational position effects in the single N levels, as for both N_0_ and N_opt_, wheat yield varied significantly with W1 > WM > W2 (data not shown).

Grains per ear and thousand grain weight were significantly higher for W1 than for W2 and WM (**Table 2**). For thousand grain weight, the significant interaction between year and crop rotational position was caused by significantly higher values for W1 compared to W2 in 2020 and 2021, but similar thousand grain weight in 2022 (**Table 2**). Ear density and harvest index were nearly the same for W1, W2, and WM (**Table 2**). Thousand grain weight and harvest index were significantly lower for 2021 than for 2020 and 2022, and in addition, harvest index was significantly higher for 2022 than for 2020 (data not shown).

**Figure 5:**
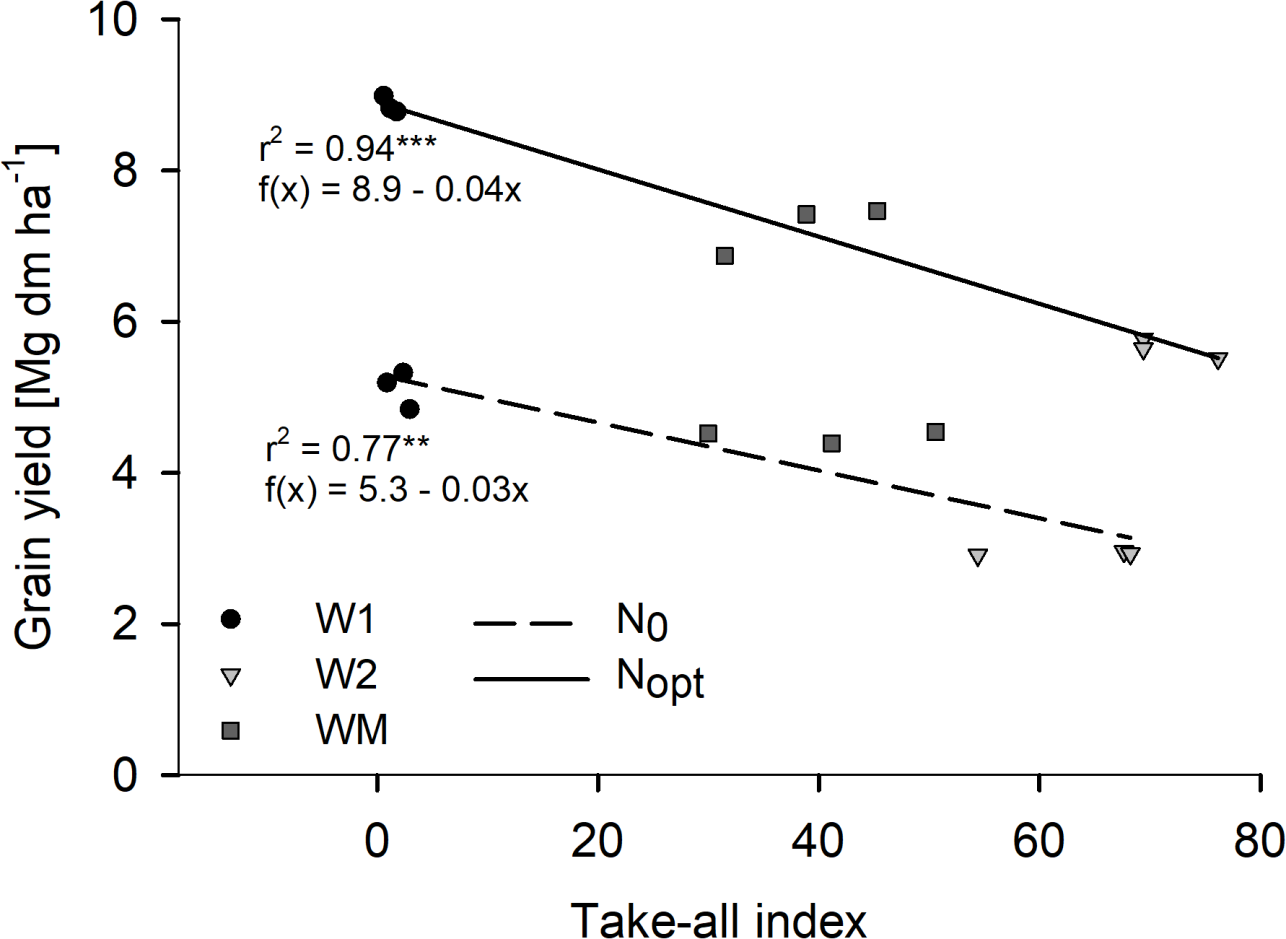
Effect of the take-all index of wheat without (N_0_) and with optimal (N_opt_) N fertilization on grain yield in Harste, 2021, *n* = 9. Asterisks indicate significant coefficient of determination at *p*< 0.01** and *p*< 0.001***. Ggt infection was confirmed with a Ggt-specific TaqMan Real-time PCR.

In 2021, take-all disease was found in all W2 and WM plots by visual determination, while no noteworthy take-all infection was found in the whole trial in 2022 (data not shown). The visually rated Ggt infection was later confirmed with a Ggt-specific TaqMan Real-time PCR analysis (data not shown). For the study year 2021, the regression analysis showed a strong negative relationship between grain yield and TAI with significant coefficients of determination for both N levels (**Figure 5**).

## Discussion

### Root growth

Across all years and sampling depths and dates, wheat RLD range (0.1–4.4 cm cm^−3^) was mostly within the range of RLD reported for cereals in other studies (0.2–2.75 cm cm^−3^, Muñoz-Romero et al., 2010; Liu et al., 2011). The crop rotational position of winter wheat had no significant effect on RLD in spring at BBCH 29; however, possible effects on RLD might have occurred in earlier phases of wheat growth in autumn and were possibly balanced out over winter. Schönhammer and Fischbeck (1987b) found in their studies that wheat after oilseed rape benefited from a lower penetration resistance and higher soil porosity. Similarly, Ball et al. (2005) suggested a modified soil structure as a reason for an effect of different pre-crops on the root system of the following crop. However, in April, no differences in soil structure in our field trial were found between the crop rotational positions investigated (Arnhold et al., 2023).

In contrast to BBCH 29, wheat RLD at BBCH 69 was significantly higher for W1 than for W2 and WM in the topsoil in the wet year 2021, which was characterized by overall twice as high RLD values across all treatments compared to the other study years. This might have been caused by the higher and more evenly distributed precipitation in 2021 compared to other years, leading to a higher soil moisture content facilitating root growth. This agrees with the results from Chen et al. (2021) who found that drought stress significantly reduced root length and root dry weight of wheat. In 2021, decreasing topsoil RLD for W2 < WM < W1 closely correlated with the occurrence of take-all disease (W2 > WM > W1). Most likely, the higher precipitation in 2021 compared to 2022 led to an increase in Ggt infection, as the fungus typically needs a warm and moist environment for development (Cook, 2003), and moreover, differences in the inoculum potential between the crop rotational positions might have developed more markedly under such conditions.

In contrast to the topsoil, subsoil RLD at BBCH 69 showed highest values in W1 in the mean over all years, which might have been caused by the taproot of oilseed rape pre-crop, allowing for a higher rooting depth and larger root system of the following crop(Perkons et al., 2014).

In the topsoil, however, weather conditions affecting soil moisture seem to be of high importance for wheat root development, potentially modifying the effect of the pre-crop and crop rotational position. However, as root samples from 0-15 cm were not analyzed at the BBCH 69 sampling date due to an exceedingly large amount of root and non-root organic material, it is not possible to specify which effect Ggt has on the RLD near the top of the wheat root system, where Ggt infection begins. Based on the data for 15–30 cm soil depth, it can be assumed that there might have been an effect of the crop rotational position in 2021, yet not in 2020 or 2022. Concerning overall crop growth, this might be of lower importance, however, since the effects of the crop rotational position on root growth in the subsoil might be more decisive. Still, in further investigations, 0–15 cm should not be excluded from root growth determination to reach a more conclusive result; otherwise, a less work-intensive analysis method like minirhizotrons or root windows might be used.

### Biomass formation during the vegetation period

Wheat aboveground biomass in BBCH stages 29, 59, and 69 was significantly higher for W1 compared to W2 and WM. Similar to the higher wheat aboveground biomass for W1, N uptake was also higher, especially at BBCH 69, which agrees with results reported by Sieling and Christen (2015) who found a significant reduction of N uptake at harvest for wheat grown after wheat compared to wheat after oilseed rape. Comparing W2 and WM in our study, no significant differences in wheat aboveground biomass occurred until harvest under N_opt_. For all crop rotational positions, a similar relative growth rate was found, showing that there were no additional differences in biomass gain between W1, W2, and WM from April until harvest, which were not already present by April. In general, the biomass development differences between W1 on the one hand and W2 and WM on the other hand during the vegetation period in our study matches the results from Sieling et al. (2005), who reported differences in wheat aboveground biomass between the first and third winter wheat after oilseed rape as break crop already at early growth stages in a field trial in northern Germany. Similar results were found by Schönhammer and Fischbeck (1987a) as well as Sieling and Christen (2015) comparing wheat after oilseed rape as break crop compared to wheat grown after wheat. Overall, a higher wheat biomass after oilseed rape as pre-crop appears to occur already in early growth stages and to be persistent until later growth stages.

### Wheat grain yield

Similar to the total aboveground biomass at harvest (BBCH 93), wheat grain yield was significantly higher for W1 than for W2 and WM. Only in 2021 did grain yield additionally vary significantly between W2 and WM, with higher values for WM. Concerning the crop rotation with W1 and W2, it can be assumed that the pre-crop is the main influence on wheat yield since, for the trial studied here, Groeneveld et al. (submitted) found that the overall crop rotation did not modify the effect of the pre-crop on wheat grain yield. Similarly, Sieling and Christen (2015) found a stronger effect of the pre-crop on grain yield of the subsequent crop compared to the overall crop rotation. The higher grain yield of wheat grown after oilseed rape agrees with the results of Weiser et al. (2018), who reported a higher grain yield of wheat grown after oilseed rape compared to wheat after wheat on commercial farms in Germany, and also with experimental results from Germany by Sieling and Christen (2015) and worldwide, as summarized in a meta-analysis of more than 900 comparisons by Angus et al. (2015). Thorup-Kristensen and Kirkegaard (2016) calculated a 0.6–0.8 Mg ha^−1^ higher grain yield of wheat grown after broad-leaf break crops compared to wheat grown after wheat, while in our study, grain yield across all years was approximately 1.7 Mg ha^−1^ higher for wheat grown after oilseed rape than for wheat grown after wheat. Differences in wheat yield between the crop rotational positions were not affected by N fertilization, which is contrary to Sieling et al. (2007) who found that increasing N fertilization increased grain yield of wheat grown after wheat more than for wheat grown after oilseed rape and thus affects the yield benefit after the latter. The yield losses of W2 and WM compared to W1 in our trial were mainly due to a significantly reduced grain number and thousand grain weight, while ear density and harvest index were not affected by the crop rotational position. Thousand grain weight and harvest index were significantly lower in 2021 than in 2020 and 2022, corresponding to the significantly lower grain yield in 2021 than in 2022. In contrast to our results, Sieling et al. (2005) found a reduced ear density of wheat following wheat and, similar to our study, a reduced thousand grain weight, but grain number was not affected. Sieling and Christen (2015) found a significant reduction of ear density, grains per ear, and thousand grain weight for wheat grown after wheat compared to oilseed rape. Thus, the relationship between yield and yield components in different crop rotational positions seems to be modified by other factors.

The negative relationship between grain yield and Ggt occurrence as previously reported by Sieling et al. (2007) was confirmed by the close correlation between grain yield and TAI found for 2021 in our study. In 2021, when a high take-all disease pressure with a different intensity for W2 and WM was found, the grain yield of WM was significantly higher than that of W2, while this was not the case in 2022, when hardly any take-all was visually determined in the trial. The higher TAI for W2 compared to WM might have been caused by the take-all decline effect in WM, resulting in a decreased disease pressure in monoculture cropping over the years due to an increased suppressiveness of the soil (Cook, 2003). As 2022 was a dry year and 2021 was a wet year, soil moisture conditions likely explain the different occurrence of Ggt disease severity in the study years (Cook, 2003). Overall, the higher TAI for W2 and WM than for W1 also corresponds well to the lower RLD for the former, which might, in turn, indicate a strong effect of RLD on wheat grain yield.

### Relationship of aboveground biomass and yield to root growth

Despite a lack of differences in rooting intensity between the different crop rotational positions early in the vegetation period, differences in aboveground biomass were found. The higher aboveground biomass for W1 at BBCH 29 corresponded to a higher N uptake, which might have been caused by a modified rhizosphere microbiome allowing for a higher nutrient acquisition per unit of root. The rhizosphere microbiome assists the wheat plants in nutrient uptake and therefore plays an important role in wheat growth as reviewed by Mahapatra et al. (2020).

The higher aboveground biomass and N uptake for W1 at BBCH 69 corresponded well to the varied RLD at the same growth stage. Similarly, Han et al. (2015) found a higher root length of spring wheat grown after taprooted chicory compared to tall fescue leading to a higher N, P, and K uptake especially at tillering. Moreover, Kirkegaard et al. (2008) reported that wheat after a break crop with a deeper and healthier root system took up more water and N from the subsoil below 1 m soil depth than wheat grown after wheat. Overall, the lower RLD for W2 and WM compared to wheat after oilseed rape can be expected to lead to a lower N uptake and a reduced biomass formation, which, in our data, is also reflected by a lower absolute growth rate for W2 and WM than for W1. Similarly high relative growth rates found for W1, W2, and WM showed that differences between biomass formation might have occurred before winter. It can thus be assumed that early root and plant development affects wheat yield. Consequently, the early plant development and root growth during the whole vegetation period should be investigated to identify processes leading to wheat yield losses for wheat cultivated after wheat.

At harvest (BBCH 93), differences in wheat aboveground biomass between WM and W2 were much more pronounced in 2021, which might have been caused by the high precipitation and take-all disease occurrence and their negative effects on RLD. In 2020 and 2022, grain yield was not significantly different between W2 and WM, which corresponds well to the absence of differences in biomass formation and RLD. The lower RLD in W2 and WM compared to W1 might have led to a lower grain number and thousand grain weight, especially in 2021. In 2020 and 2021, grain yield was on an identical level, which is in contrast to the differences in RLD between 2020 and 2021. Similarly, in all crop rotational positions, grain yield was highest in the driest year 2022, despite a generally lower RLD than in 2021. Thus, other factors seem to modify the effect of RLD on yield.

In 2022, the higher grain yield for W1 despite a lack of differences in top- and subsoil RLD between the crop rotational positions might have been caused by a shift in the rhizosphere microbiome in the topsoil and a higher or more efficient nutrient acquisition. For example, Qu et al. (2020) investigated two different *Pseudomonas* genera of rhizobacteria and found a beneficial effect on N uptake and grain yield of wheat under greenhouse conditions. In Washington State, Ggt-suppressive soils are characterized by an increase of populations of antagonistic *Pseudomonas* spp., as reported by Weller et al. (2002). As the relationship between roots and rhizosphere microbiome is highly complex and thousands of bacteria genera are involved, further research is necessary to identify rhizobacteria that promote wheat biomass formation and final grain yield (Rascovan et al., 2016; Mahapatra et al., 2020). Possibly, with a beneficial rhizosphere microbiome, a lower RLD is sufficient to obtain a higher aboveground biomass and final grain yield.

To sum up, subsoil RLD of winter wheat was higher after oilseed rape as pre-crop compared to winter wheat as pre-crop at a later growth stage, which corresponded to a higher wheat biomass and final grain yield. Root length density of winter wheat, particularly in the subsoil, might be the key to understand wheat biomass formation and grain yields in different crop rotational positions. Further investigations are needed to identify by which mechanisms pre-crops affect root growth of the following crop and the related rhizosphere processes, especially in the very early development phase.

## Conclusion

In our study, a higher RLD correlated with a higher N uptake and a higher biomass production and grain yield for wheat grown after oilseed rape compared to wheat grown after wheat. Differences in biomass formation developed already at early growth stages and were persistent during the vegetation period. Yield differences correlated well with take-all occurrence in a wet year, but as differences in grain yield between crop rotational positions also occurred in dryer years without visible take-all disease, an effect of the crop rotational position beyond take-all disease pressure like a shift in the rhizosphere microbiome can be assumed. Wheat root length density, particularly in the subsoil, might be the key to understand wheat biomass formation and grain yields in different crop rotational positions. Further studies are needed to investigate the rhizosphere microbiome and the related processes as well as water and nutrient uptake in detail. In addition, it should be investigated if the effect of the crop rotational position on wheat development is modified by Ggt non-host crops other than oilseed rape and the choice of the wheat genotype.

## Data availability statement

The raw data supporting the conclusions of this article will be made available by the authors, without undue reservation.

## Author contributions

JA: Writing – original draft, Conceptualization, Data curation, Formal Analysis, Investigation, Visualization, Writing – review & editing. DG: Writing – review & editing, Conceptualization, Formal Analysis, Investigation. AB-K: Writing – review & editing, Investigation. H-JK: Conceptualization, Funding acquisition, Investigation, Project administration, Supervision, Writing – review & editing.
