# Correction: Effect of crop rotational position and nitrogen supply on root development and yield of winter wheat

**DOI:** 10.3389/fpls.2025.1673401

**Published:** 2025-09-04

**Authors:** Jessica Arnhold, Dennis Grunwald, Andrea Braun-Kiewnick, Heinz-Josef Koch

**Affiliations:** ^1^ Department of Agronomy, Institute of Sugar Beet Research, Göttingen, Germany; ^2^ Institute for Epidemiology and Pathogen Diagnostics, Julius Kühn-Institute - Federal Research Centre for Cultivated Plants, Braunschweig, Germany

**Keywords:** root length density, grain yield, nitrogen uptake, biomass, oilseed rape, take-all disease

There was a mistake in **Figure 2**, page 6 as published. The values for root length density in 30–120 cm were erroneously calculated as sum and not as mean of three separately analysed soil depths (30-60, 60–90 and 90–120 cm). The corrected **Figure 2** appears below.

**Figure 2:**
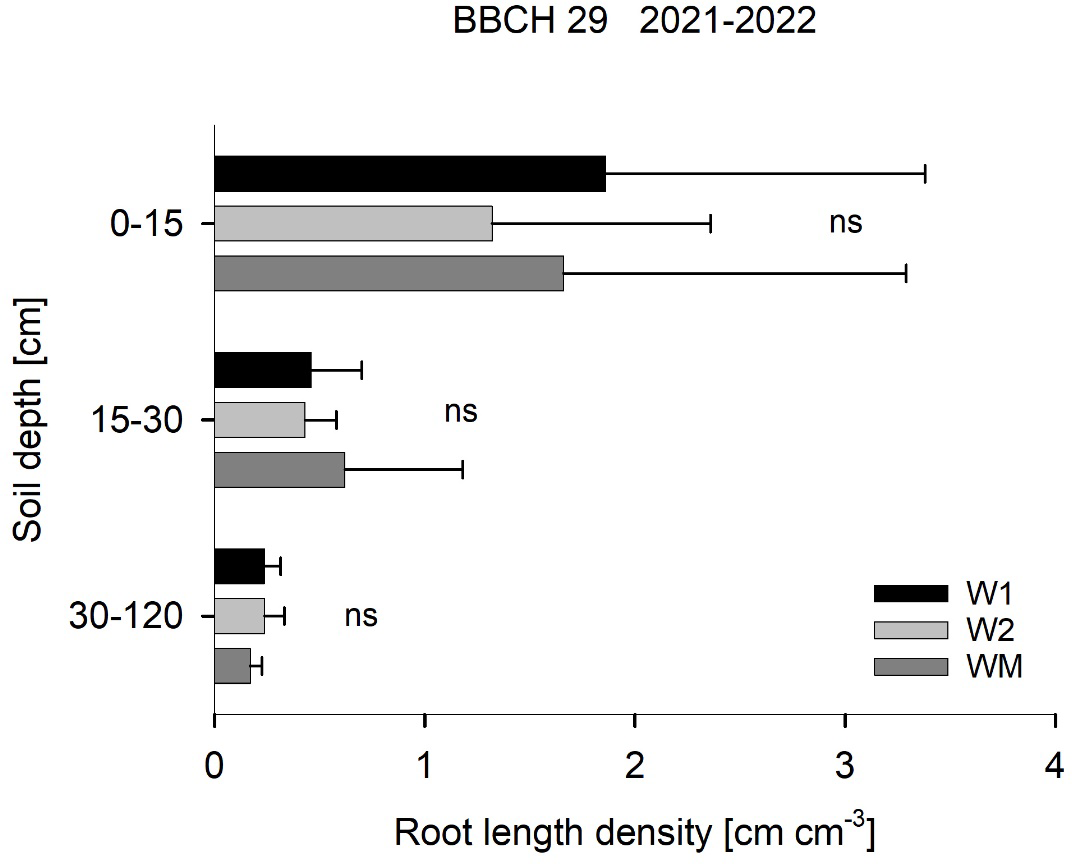
Effect of the crop rotational position of winter wheat (W1 = first wheat after oilseed rape, W2 = second wheat after oilseed rape, WM = wheat monoculture) on root length density in three soil depths at BBCH 29 in Harste, data from 2021 and 2022, n = 12. Bars show means with standard deviation, ns, not significant (p ≥ 0.05).

There was a mistake in **Figure 3**, page 6 as published. The values for root length density in 30–120 cm were erroneously calculated as sum and not as mean of three separately analysed soil depths (30-60, 60–90 and 90–120 cm). Additionally, the values for root length density in 30–120 cm in 2022 were erroneously copied from 2020. The corrected **Figure 3** appears below.

**Figure 3:**
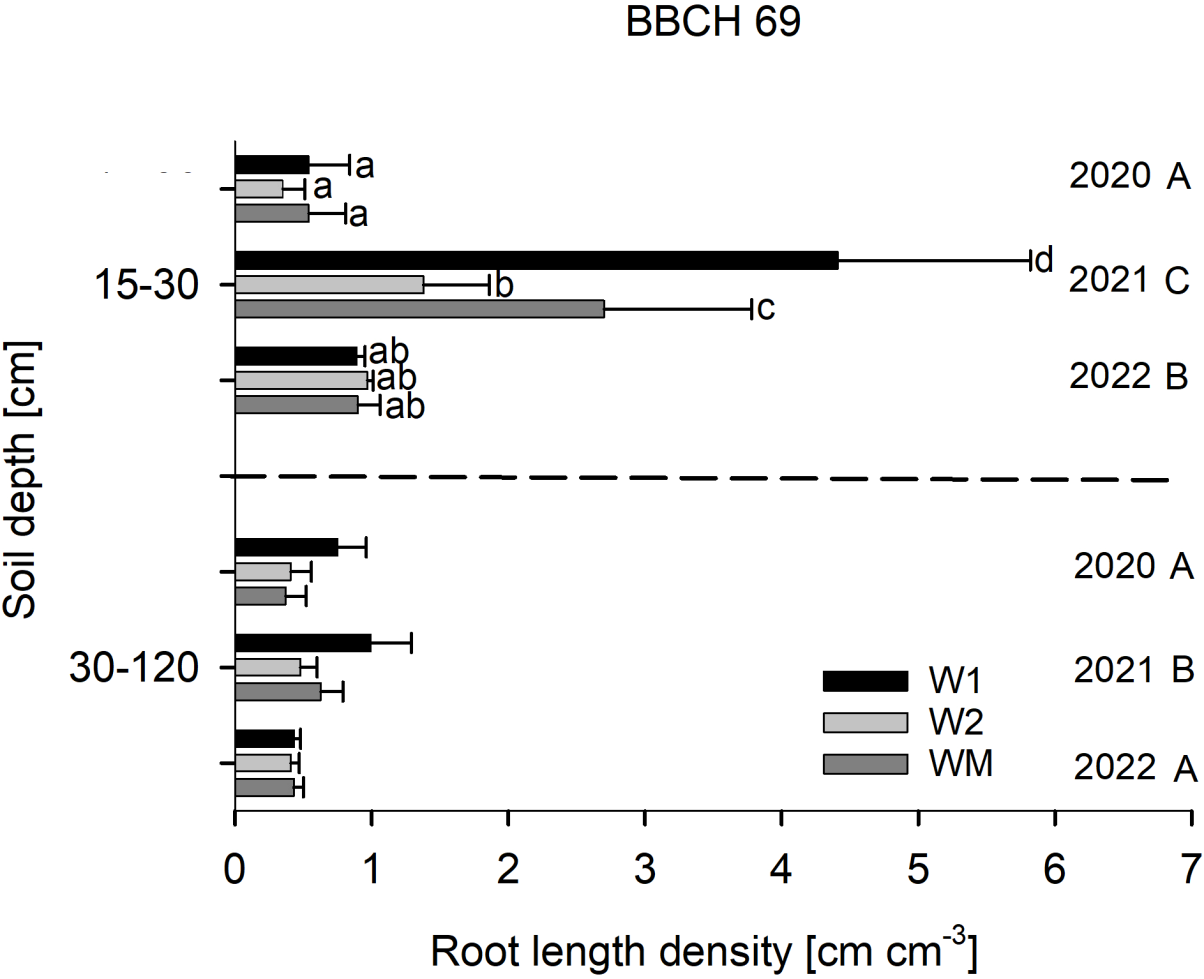
Effect of the crop rotational position of winter wheat (W1 = first wheat after oilseed rape, W2 = second wheat after oilseed rape, WM = wheat monoculture) on root length density (RLD) in two soil depths at BBCH 69 in Harste, n = 6, mean across N fertilization. Bars show means with standard deviation. Lowercase letters show significant differences between means in each depth and capital letters show significant differences between means of each year (p < 0.05). RLD varied significantly with W1 (0.7 ± 0.3 cm cm^−3^) > WM (0.5 ± 0.2 cm cm^−3^) and W2 (0.4 ± 0.1 cm cm^−3^) in 30 -120 cm soil depth.

There was a mistake in the caption of **Figure 3**, page 6 as published. The values mentioned in the last sentence (“RLD varied significantly with W1 (2.2 ± 0.9 cm cm^−3^) > WM (1.4 ± 0.5 cm cm^−3^) and W2 (1.3 ± 0.3 cm cm^−3^) in 30–120 cm soil depth.”) were erroneously calculated as sum and not as mean of three separately analysed soil depths (30-60, 60–90 and 90–120 cm).

The corrected caption of **Figure 3** appears below:

“Effect of the crop rotational position of winter wheat (W1 = first wheat after oilseed rape, W2 = second wheat after oilseed rape, WM = wheat monoculture) on root length density (RLD) in two soil depths at BBCH 69 in Harste, n = 6, mean across N fertilization. Bars show means with standard deviation. Lowercase letters show significant differences between means in each depth and capital letters show significant differences between means of each year (p < 0.05). RLD varied significantly with W1 (0.7 ± 0.3 cm cm^−3^) > WM (0.5 ± 0.2 cm cm^−3^) and W2 (0.4 ± 0.1 cm cm^−3^) in 30–120 cm soil depth.”

In the **Abstract**, an erroneous statement based on the above-mentioned errors has been made in the last part of the sentence “Subsoil root length density of winter wheat was significantly higher after oilseed rape as pre-crop than after wheat, which was independent of take-all occurrence.”

The corrected sentence is below:

“Subsoil root length density of winter wheat was significantly higher after oilseed rape as pre-crop than after wheat.”

A correction has been made to the **Results** section, third paragraph:

“In 30–120 cm soil depth at BBCH 69, RLD varied significantly with W1 (0.7 ± 0.3 cm cm^−3^) > WM (0.5 ± 0.2 cm cm^−3^) and W2 (0.4 ± 0.1 cm cm^−3^) across all study years (**Figure 3**).”

A correction has been made to the **Discussion** section, first paragraph:

“Across all years and sampling depths and dates, wheat RLD range (0.1–4.4 cm cm^−3^) was mostly within the range of RLD reported for cereals in other studies (0.2–2.75 cm cm^−3^, Muñoz-Romero et al., 2010; Liu et al., 2011).”

A correction has been made to the **Discussion** section, subsection *Root Growth*, third paragraph:

“In contrast to the topsoil, subsoil RLD at BBCH 69 showed highest values in W1 in the mean over all years, which might have been caused by the taproot of oilseed rape pre-crop, allowing for a higher rooting depth and larger root system of the following crop (Perkons et al., 2014).”

A correction has been made to the **Discussion** section, subsection *Relationship of aboveground biomass and yield to root growth*, fourth paragraph:

“In 2022, the higher grain yield for W1 despite a lack of differences in top- and subsoil RLD between the crop rotational positions might have been caused by a shift in the rhizosphere microbiome in the topsoil and a higher or more efficient nutrient acquisition.”

A correction has been made to the **Discussion** section, subsection *Relationship of aboveground biomass and yield to root growth*, fifth paragraph:

“To sum up, subsoil RLD of winter wheat was higher after oilseed rape as pre-crop compared to winter wheat as pre-crop at a later growth stage, which corresponded to a higher wheat biomass and final grain yield.”

The original version of this article has been updated.

